# NEIGHBORHOOD TRANSPORTATION OPTIONS AND TRANSITIONS TO NONDRIVING

**DOI:** 10.1093/geroni/igae098.2574

**Published:** 2024-12-31

**Authors:** Kellia Hansmann, Carolyn McAndrews, Ronald Gangnon, Stephanie Robert

**Affiliations:** University of Wisconsin School of Medicine and Public Health, Madison, Wisconsin, United States; University of Wisconsin Madison, Madison, Wisconsin, United States; University of Wisconsin Madison, Madison, Wisconsin, United States; University of Wisconsin Madison, Madison, Wisconsin, United States

## Abstract

Many older drivers rely on their social networks for transportation support as they transition to non-driving, but still experience barriers to maintaining the mobility they had when driving independently. In the transition to non-driving, older adults also need transportation options in their neighborhoods to maintain mobility. We examined whether neighborhood transportation options were associated with changes in driving behaviors among U.S. older drivers. We analyzed National Health and Aging Trends Study data from community-dwelling drivers (n = 5,102; 2015 cohort). We used weighted logistic regression models to estimate whether walking or taking public transit was associated with the number of driving behaviors they avoided (avoided driving at night, in bad weather, on highways, or alone) at the three-year follow up. We also estimated the association between the National Walkability Index (NWI) and driving reduction at the three-year follow up. In 2015, 52% of older drivers reported walking to get places outside their homes, but few reported taking public transit (6%). In adjusted models, we found no associations between walking or taking public transit to get places in 2015 and driving reduction over a three-year period. However, older drivers living in very walkable neighborhoods in 2015 had greater odds of driving avoidance behaviors compared to those living in very unwalkable neighborhoods (NWI of 18 vs. 2 out of 20: adjusted odds ratio = 1.73; 95% Confidence Interval: 1.14-2.63). Future research should examine what modifiable aspects of neighborhood transportation options might play a role in supporting transitions to non-driving for older drivers.

